# Ulcer-like projections into the dilated false lumen after stent-graft placement for aortic dissection: A case report

**DOI:** 10.1097/MD.0000000000028472

**Published:** 2022-01-07

**Authors:** Xi He, Eijun Sueyoshi, Shun Nakaji, Masataka Uetani

**Affiliations:** a Department of Radiological Sciences, Graduate School of Biomedical Sciences, Nagasaki University, Sakamoto, Nagasaki, Japan; b Department of Cardiovascular Surgery, Nagasaki University Hospital, 1-7-1 Sakamoto, Nagasaki, Japan.

**Keywords:** aortic dissection, computed tomography, thoracic endovascular aortic repair, ulcer-like projection

## Abstract

**Rationale:**

Acute type A aortic dissection and chronic type B aortic dissection (TBAD) occurs simultaneously in rare cases. Although the development of ulcer-like projection (ULP) is associated with an increase in adverse aorta-related events, the false-lumen enlargement caused by the ULP progression is uncommon.

**Patient concerns:**

A 72-year-old female with chronic TBAD was admitted to our unit with back and chest pain. Computed tomography revealed acute type A aortic dissection and a hematoma caused by rupturing of the descending aorta due to chronic TBAD. After endovascular intervention, the false lumen thrombosed and shrunk.

**Diagnosis:**

After 9 months, a developing ULP, which projected into a dilating false lumen, was found. An impending ruptured descending aortic aneurysm was confirmed.

**Interventions:**

Emergency Total arch replacement and thoracic endovascular aortic repair (TEVAR) was performed.

**Outcomes:**

The procedure was successful. One year later, regular follow-up showed that the false lumen had completely shrunk.

**Lessons:**

ULP can arise and cause progressive dilation of false lumen after TEVAR. Careful and regular computed tomography examinations are required for early diagnosis of false lumen becoming thrombosed after TEVAR. Close follow-up and timely intervention, including TEVAR, should be considered in cases of aortic enlargement due to a newly developed ULP.

## 1 Introduction

Thoracic endovascular aortic repair (TEVAR) is one of the conventional treatments for aortic dissection (AD). TEVAR can effectively shrink the false lumens of Stanford type B AD (TBAD).^[1,2]^ Ulcer-like projections (ULP) can sometimes arise in cases of AD involving thrombosed false lumens.^[3–8]^ The definition of ULP remains unclear, and they can include intimal tears, occlusive lesions, penetrating atherosclerotic ulcers, and detached aortic branch orifices.^[9]^ Miyahara et al^[10]^ reported that ULP appear as localized blood-filled pouches that protrude from the true aortic lumen into the thrombosed false lumen on radiological imaging. Here, we present a case of false lumen dilation due to a ULP that developed after TEVAR. To the best of our knowledge, this is the first report of this condition.

## 2 Case report

**Figure 1:**
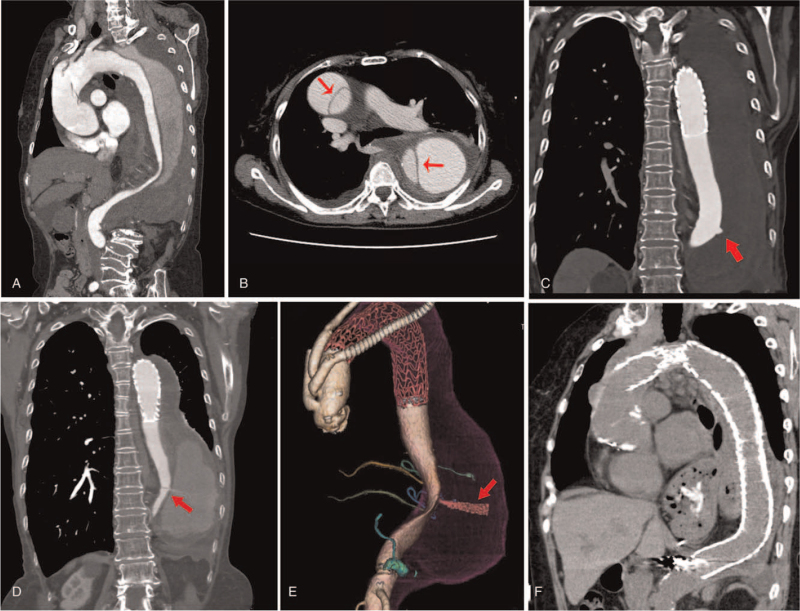
Contrast-enhanced CT performed on admission. A. Acute type A dissection of the ascending aorta and type B aortic dissection surrounded by a hematoma. B. Axial view showed the occurrence of TAAD and TBAD at the same time (arrow). C. Coronal view showed the thrombosed false lumen and a new aortic ulcer-like change (arrow) in 1-month follow-up. D. Coronal view showed the dilated false lumen and jet-like flow from the developing ulcer-like projection (arrow). E. Volume-rendered chest CT angiographic image showed jet-like flow (arrow) into the enlarged false lumen. F. Follow-up CT performed 1 year after the endovascular intervention and the image showed a correctly positioned endograft with a thrombotic and shrunken false lumen. CT = computed tomography, TAAD = type A aortic dissection, TBAD = type B aortic dissection.

A 72-year-old female had been diagnosed with chronic TBAD 9 years earlier and had undergone regular follow-up instead of interventions at our hospital. She was admitted to the emergency care unit because of back and chest pain. After a cardiological evaluation excluded coronary artery disease, contrast-enhanced computed tomography (CT) revealed acute type A AD of the ascending aorta and a hematoma caused by chronic TBAD-induced rupturing of the descending thoracic aorta (Fig. 1A and B). The type A AD and chronic TBAD-induced rupture occurred at the same time. The ascending aorta was replaced with an artificial vessed and the distal dissection was treated with TEVAR. TEVAR was performed using a conformable GORE TAG thoracic endoprosthesis (W. L. Gore & Associated, Inc., Flagstaff, AZ) (TGU313115J). Coil embolization of the reentry site was also performed using fibered IDC coils (Boston Scientific, Marlborough, MA) [(12 mm × 30 cm) × 1 and (8 mm × 20 cm) × 2], Nester microcoils (Cook Medical Inc., Bloomington, IN) [(4 mm × 14 cm) × 8], and 1.5 mL 25% N-butyl-2-cyanoacrylate. At the 1-month follow-up, the false lumen was fully thrombosed and gradually reduced in diameter.However, CT showed an ulcer-like change in the wall of the true lumen of the descending aorta (Fig. 1C). Long-term CT monitoring was scheduled, which showed progressive expansion of the false lumen. After 8 months and 2 weeks, the patient was referred to our unit due to the onset of severe back pain. Contrast-enhanced CT and volume-rendered chest CT showed a dilated false lumen with a jet of blood flow from the developing ULP into the intramural hematoma, which were suggestive of an impending rupture (Fig. 1D and E, and see Video S1, Supplemental Digital Content, which demonstrates the jet-like flow of blood from the progressive ULP). An additional stent graft was placed for the expanded false lumen immediately using a conformable GORE TAG (TGU313115J). The procedure was successful, and the patient was discharged on the 9^th^ postoperative day. One year later, regular follow-up showed that the false lumen had completely shrunk (Fig. 1F). This study was approved by the institutional review board of Nagasaki University Hospital. Informed consent was obtained from the patient.

## 3 Discussion

A previous study demonstrated that patients with ULP tended to exhibit poor survival because of late aorta-related events, including aortic rupturing.^[10–12]^ This was specified that a third of ULP was found to progress to aortic enlargement, with the mean increase in aortic diameter from onset being 15% (range, 5%–45%).^[7]^ In the current case, the newly developed ULP caused progressive dilation of false lumen after TEVAR, and it finally led to an impending rupture of the descending aorta. Regarding the pathogenesis, ulcer-like change in the wall of the true lumen is regarded to involve direct flow communication between false and true lumens.^[13]^ Some reports indicated that it represented the site of reentry tear, which is secondary to the intramural hematoma expansion followed by bleeding from the medial layer. However, the intimal tear without blood flow was also observed even at the early stage and the incidence of new intimal disruption did not increase over time.^[14]^ It is possible that intimal defects or tears exist at the onset of intramural hematoma as a“microtear” or small intimal communication in the closed and thrombosed false lumen. These new intimal disruptions often progressed in size as ULP does, thus, the existence as a distinct pathological entity of ULP may be fully demonstrated by the initial “microtear”.^[15]^

Kitai et al^[15]^ reported that the development of ULP were found in 36% patients with TBAD with thrombosed false lumens within 30 days after discharge. Furthermore, ULP was associated with poor prognosis as it frequently enlarged and progressed to aneurysms, which required timely surgery or percutaneous aortic repair.^[7]^ The ULP in present case, located in the descending aorta, was identified together with a thrombosed false lumen at 3 months after stent graft. Then, it projected into a dilated false lumen, resulting in the potential rupture of a thoracic aortic aneurysm. Considering the progressive course of ULP, endovascular intervention or prophylactic surgery may be more promising than conservative medical treatment, especially the repair targeting the intimal plaque rupture site.^[14]^ Follow-up imaging is necessary since the development of ULP can be identified based on the thrombolysis of superficial thrombi.^[15]^ The observation of intimal defects or ULP may help to predict aorta-related events and assess the necessity of prophylactic stent-grafting.

In this study, ULP can arise and cause progressive dilation of false lumen after TEVAR. Careful and regular CT examinations are required if a false lumen becomes thrombosed after TEVAR. Close follow-up and timely intervention, including TEVAR, should be considered in cases of aortic enlargement due to a newly developed ULP.

## Author contributions

**Conceptualization:** Eijun Sueyoshi, Shun Nakaji.

**Data curation:** Xi He, Eijun Sueyoshi.

**Formal analysis:** Xi He.

**Investigation:** Xi He.

**Project administration:** Masataka Uetani.

**Resources:** Shun Nakaji.

**Supervision:** Masataka Uetani.

**Validation:** Eijun Sueyoshi, Masataka Uetani.

**Writing – original draft:** Xi He.

**Writing – review & editing:** Eijun Sueyoshi, Masataka Uetani.

## Supplementary Material

Supplemental Digital Content
